# Environmental effects on efficacy of herbicides for postemergence goosegrass (*Eleusine indica*) control

**DOI:** 10.1038/s41598-020-77570-5

**Published:** 2020-11-25

**Authors:** Avat Shekoofa, James T. Brosnan, Jose J. Vargas, Daniel P. Tuck, Matthew T. Elmore

**Affiliations:** 1 Department of Plant Sciences, University of Tennessee at Knoxville, 2431 Joe Johnson Drive, Knoxville, TN 37996 USA; 2 Department of Plant Biology, Rutgers, The State University of New Jersey, 59 Dudley Road, New Brunswick, NJ 08901 USA

**Keywords:** Plant stress responses, Plant sciences, Environmental sciences

## Abstract

Experiments were conducted to understand environmental effects on efficacy of herbicides used to control goosegrass (*Eleusine indica* L. Gaertn.). Herbicides were applied to goosegrass maintained at soil moisture contents (VMC) of < 12%, 12 to 20%, or > 20%. Herbicides included fenoxaprop-p-ethyl (140 g ha^−1^), topramezone (25 g ha^−1^), foramsulfuron (44 g ha^−1^), 2,4-D + dicamba + MCPP + carfentrazone (860 + 80 + 270 + 28 g ha^−1^), and thiencarbazone-methyl + foramsulfuron + halosulfuron-methyl (22 + 45 + 69 g ha^−1^). Goosegrass control increased as VMC increased. Vapor pressure deficit (VPD) and air temperature were manipulated to determine effects of evaporative demand on foramsulfuron. Effects of soil drying were also studied following foramsulfuron application. Reductions in transpiration rate (TR) and leaf area were greatest with foramsulfuron applications to goosegrass in silt-loam under high evaporative demand (3 kPa VPD, 38 °C). Foramsulfuron had no effect on goosegrass in silica-sand regardless of evaporative demand. TR dropped to 0.2 mmh^−1^ within eight days after application to goosegrass in silt-loam compared to 18 days in silica-sand. Overall, foramsulfuron efficacy on goosegrass was maximized under conditions of high soil moisture and evaporative demand, and may be reduced in sandy soils that hold less water.

## Introduction

Goosegrass (*Eleusine indica* L. Gaertn.) is a widely distributed weed of many agricultural systems. Goosegrass tolerates low soil oxygen^1^ and is particularly problematic in turfgrass systems, often found on golf courses and sports fields in soils subjected to compaction from foot or vehicular traffic. Turfgrass canopy cover is often reduced in these environments, which can favor goosegrass competition^2^. Tillered goosegrass plants exhibit a bunch type growth habit that disrupts surface uniformity leading to compromised ball roll and footing during sport. An abundant seed producer, one goosegrass plant can produce upwards of 140,000 seeds that germinate under conditions of fluctuating (day/night) air temperature and light^3,4^.

Considering that there are no known cultural means of controlling goosegrass in a perennial turfgrass sward^5^, the species is often targeted with extensive use of pre- or postemergence herbicides. This has resulted in selection of herbicide-resistant goosegrass with the species evolving resistance to eight sites of action^6^. As one of the top 15 herbicide-resistant weed species, there are confirmed cases of goosegrass resistance in turfgrass to herbicidal inhibitors of cellular mitosis and protoporphyrinogen oxidase applied preemergence^7,8^, as well as postemergence applications of photosystem II and acetyl co-A carboxylase (ACCase) inhibitors^9,10^.

Yamaji et al.^11^ indicated that environmental factors such as soil properties, rainfall, and field preparation affect performance of postemergence herbicides. For example, efficacy of postemergence herbicides for annual grass control under soil moisture deficit are well documented. Dortenzio and Norris^12^ reported less control of barnyardgrass [*Echinochloa crus-galli* (L.) Beauv] and wild oat (*Avena fatua* L.) with the ACCase-inhibiting herbicide diclofop-methyl (HRAC Group #1) applied to plants maintained at 10% soil moisture compared to 30%. Interestingly, the researchers observed that increasing soil moisture from 10 to 30% during the first 4 d after application resulted in barnyardgrass control similar to treating plants maintained at 30% soil moisture. Reducing soil moisture during the first 24 h after application also significantly reduced barnyardgrass control with diclofop-methyl leading the researchers to conclude that irrigation should be applied 1 to 2 d after diclofop-methyl application for maximum effectiveness. Boydston^13^ noted 40% reductions in green foxtail [*Setaria viridis* (L.) Beauv.] control with fenoxaprop-ethyl, fluazifop-butyl, and haloxyfop-methyl (all HRAC Group #1) when moisture was withheld before or after treatment. Kidder and Behrens^14^ documented that the ACCase-inibiting herbicide haloxyfop-methyl was less injurious to green foxtail and proso millet (*Panicum miliaceum* L.) when applied to plants growing under moisture stress. The researchers illustrated that moisture stress reduced retention of herbicide applied to proso millet leaves and reduced herbicide translocation in both species. Reynolds et al.^15^ reported reduced basipetal translocation of ^14^C-fluazifop-butyl, ^14^C-sethoxydim, ^14^C-haloxyfop-methyl, and ^14^C-quizalofop-ethyl applied to grain sorghum [*Sorghum bicolor* (L.) Moench] under moisture stress (leaf water potential − 680 kPa). Reduced control of barnyardgrass was reported with applications of diclofop-methyl to plants maintained at low water potential as well^16^. When studying wild accessions of goosegrass and plantain signalgrass (*Urochloa plantaginea*) from Brazil, efficacy of ACCase-inhibiting herbicides was reduced with applications made to plants under moisture stress^17,18^. Increased expression of superoxide dismutase and catalase under drought conditions have been linked with reduced phytotoxicity following fenoxaprop-ethyl treatment to wild oat^19^.

Data describing effects of soil moisture stress on postemergence herbicides other than ACCase-inhibitors are limited. However, Olson et al.^20^ reported that cheatgrass (*Bromus tectorum* L.) and wild oat were injured more from sulfosulfuron (HRAC Group #2) applied when soil moisture was at saturation compared with one-third moisture content. Additionally, the researchers reported greater efficacy when air temperature was 25/23 °C after application compared to 5/3 °C. Aghabeigi and Khodadadi^21^ demonstrated that moisture stress reductions in efficacy of clodinafop-propargyl and mesosulfuron-methyl could be mitigated via increases in application rate. Fausey and Renner^22^ showed that increasing temperature from 10 to 40 °C increased CGA-248757 (fluthiacet-methyl; HRAC Group #14) and flumiclorac (HRAC Group #14) activity on redroot pigweed (*Amaranthus retroflexus* L.) by 68 and 60%, respectively. In turfgrass, elevated relative humidity has been reported to increase activity of mesotrione (HRAC Group #27) applied to smooth crabgrass (*Digitaria ischaemum* Schreb. Ex. Muhl.) in laboratory studies^23^.

Herbicides from several mode-of-action groups are labeled for postemergence goosegrass control in turfgrass systems including fenoxaprop (HRAC Group #1), foramsulfuron (HRAC Group #2), topramezone (HRAC Group #27), as well as pre-packaged mixtures of thiencarbazone-methyl + foramsulfuron + halosulfuron (all HRAC Group #2 herbicides) and carfentrazone + 2,4-D + MCPP + dicamba (HRAC Group #14 + three active ingredients in HRAC Group #4). Investigations pertaining to the effects of moisture stress on the efficacy of herbicides other than ACCase inhibitors (HRAC Group #1) are limited. Understanding impacts of environmental factors on efficacy of these herbicides would assist turfgrass managers with goosegrass management decisions in the field. To that end, we hypothesized that environmental factors such as moisture stress, air temperature, and evaporative demand affect the efficacy of several herbicides used for postemergence goosegrass in turfgrass, particularly inhibitors of acetolactate synthase (HRAC Group #2). A series of glasshouse and growth chamber experiments were conducted to generate the data needed to confirm or reject this hypothesis.

## Materials and methods

A series of glasshouse and growth chamber experiments were conducted in 2018 to 2019 to understand the effects of several environmental factors on the efficacy of various postemergence herbicides labeled for goosegrass control in managed turfgrass systems such as golf courses, sports fields, and lawns.

### Soil moisture effects on herbicide efficacy

A glasshouse experiment was conducted to determine the effect of volumetric soil moisture content on goosegrass control efficacy with several postemergence herbicides. The experiment was initiated on 5 November 2018 at the University of Tennessee (Knoxville, TN; 35.56°N, 83.56°W) and repeated on 22 July 2019 at Rutgers University (New Brunswick, NJ; 40.28°N, 74.26°W). Maximum/minimum air temperatures in the glasshouse averaged 25/21 °C in 2018 and 35/19 °C in 2019.

Goosegrass was surface seeded into greenhouse pots filled with field soil (1050 cm^3^ volume) amended with calcined clay (Turface. Profile Professional Products. Buffalo Grove, IL) in a 60:40 soil:clay ratio by volume. In 2018, soil was a Sequatchie silt loam (fine-loamy, siliceous, semiactive, thermic humic Hapludult) measuring pH 6.2. In 2019, soil was a Holmdel sandy loam (fine-loamy, mixed, active, mesic Aquic Hapludults) measuring pH 6.3. Pots were supplied with complete fertilizer (20 N—8.7 P—16.6 K; Howard Johnson’s Triple Twenty Plus Minors. Milwaukee, WI) at 49 kg N ha^−1^ wk^−1^ and irrigated to promote seed establishment. Pots were hand-thinned to contain three goosegrass plants, each with a minimum of three-tillers. After thinning, pots were divided into groups (for the remainder of the experiment) based on volumetric soil moisture content (VMC): < 12%, 12 to 20%, or > 20%. These ranges were selected to represent soil moisture conditions common in maintained turfgrass systems such as golf courses, sports fields, and lawns. Volumetric soil moisture content in each pot was monitored daily using a moisture meter (ML-3 Theta Probe. Delta-T Devices. Cambridge, United Kingdom). Corrective irrigation was applied as needed when a pot measured outside of an intended VMC target. Goosegrass plants were allowed three weeks to acclimate to the different VMC regimes before applying herbicide treatments.

Herbicides included in this experiment were as follows: carfentrazone-ethyl + 2,4-D-ester + mecoprop-p + dicamba (Speedzone. PBI Gordon Corporation. Shawnee, KS) at 28 + 857 + 269 + 78 g ha^−1^, respectively; topramezone (Pylex. BASF Corporation. Research Triangle Park, NC) at 24 g ha^−1^; fenoxaprop (Acclaim Extra. Bayer Crop Science. Cary, NC) at 140 g ha^−1^; foramsulfuron (Revolver. Bayer Crop Science. St. Louis, MO) at 44 g ha^−1^; and thiencarbazone-methyl + foramsulfuron + halosulfuron-methyl (Tribute Total. Bayer Crop Science. St. Louis, MO) at 22 + 45 + 67 g ha^−1^, respectively. Adjuvants were included with herbicides per label recommendations. Herbicides were applied in an enclosed spray chamber (Generation III track sprayer. DeVries Manufacturing, Hollandale, MN) using a water carrier at 215 L ha^−1^ in 2018 and 440 L ha^−1^ in 2019 through an 8004 EVS nozzle (TeeJet, Wheaton, IL). Non-treated controls (for each VMC regime) were included for comparison. Each year goosegrass control was visually assessed using a 0 (i.e., lowest) to 100% (i.e., highest) scale relative to non-treated controls at 36 days after treatment (DAT).

Treatments were arranged as a 3 × 6 factorial (i.e., three VMC regimes and six herbicide treatments), randomized complete block design, with six replications. All data were subjected to analysis of variance in R^24^ using expected means squares of McIntosh^25^ in the ‘agricolae’ package^26^. Means were separated using Fisher’s protected least significant difference test (α = 0.05) via the ‘LSD.test’ function in R.

### Vapor pressure deficit and air temperature effects

A series of walk-in growth chamber experiments were conducted to determine effects of vapor pressure deficit (VPD) on foramsulfuron applied to multi-tiller goosegrass at varying air temperatures. Effects of VPD were determined under 12-h photoperiod and day/night air temperatures of 32/26 °C, as well as 38/26 °C. The photosynthetic photon flux density in the growth chamber was 550 to 600 µmol m^−2^ s^−1^ during all experiments. All experiments were repeated in both time and space during summer 2019.

Goosegrass plants were grown in pots constructed from polyvinyl chloride pipe (10-cm diameter) in a greenhouse at West Tennessee Research and Education Center (WTREC; Jackson, TN). Each pipe was cut to a length of 25-cm with the bottom of each pipe fitted with a flat-end cap in which a small hole was drilled to allow for drainage of excess water. Pots were filled with Sequatchie silt loam soil (fine-loamy, siliceous, semiactive, thermic humic Hapludult), measuring pH 6.2, or silica sand. Goosegrass was surface seeded into these pots, irrigated daily, and maintained at day/night air temperatures of 32/26 °C in the greenhouse until maturing to a three-tiller growth stage. Once the growth stage was reached, goosegrass plants were moved outside of the greenhouse for application of foramsulfuron (Revolver. Bayer CropScience. St. Louis, MO) at 0 or 44 g ha^−1^ using a CO_2_-pressurized calibrated to deliver 374 L ha^−1^. Immediately following the application both treated (44 g ha^−1^) and non-treated (0 g ha^−1^) plants were placed in the walk-in chamber. In all experiments there were five replications of each foramsulfuron treatment (0 or 44 g ha^−1^) per soil type.

Transpiration rate was assessed 4 and 14 days after foramsulfuron treatment (DAT) as a measure of herbicidal activity. The following protocol was used to monitor transpiration rate (TR) in all experiments. The evening before TR measurements, pots were over-watered and allowed to drain overnight. The soil surface around each plant was sealed with aluminum foil to prevent soil evaporation. The following morning, aboveground vegetation of each goosegrass plant was enclosed in a 21-L clear container to measure TR across a range of VPDs. A toilet flange attached to the top of each previously described pot allowed for the attachment of a VPD chamber. Each chamber was fitted with a 12 V, 80 mm diameter cooling fan (Masscool, Fanner Tech USA) to continuously stir the air inside the chamber. Stirring of the air helped to maintain plant temperature near ambient air temperature within the VPD chamber. A humidity/temperature data logger (Lascar Electronics, Erie, PA) was mounted through the side-wall of each chamber to monitor environmental conditions inside the chamber.

Transpiration rates were measured for each goosegrass plant at three humidity levels using previously published methods^27,28^: low VPD (0.5 to 1.5 kPa), medium VPD (1.5 to 2.5 kPa), and high VPD (2.5 to 4.0 kPa). Target humidity levels inside the VPD chambers were obtained by adjusting air-flow rate; in the case of the highest VPD treatment, the air was also initially flowed through a column of silica gel to aid in moisture removal. Conditions in the VPD chambers were allowed to stabilize for 30 min after setting each humidity level and then the entire pot-chamber unit was weighed to obtain an initial weight. The plants were then exposed for 1 h to each humidity level and reweighed. Transpiration rate data were collected for 48 h. Leaf area of each goosegrass plant was measured and calculated non-destructively 4 DAT using Eq. (1), where the value of the constant (A) is 0.75^29^.

1 $${\text{Leaf Area}} = {\text{Length}}*{\text{Width}}*{\text{A}}$$

At 14 DAT, goosegrass leaves were dissected and the total area of the leaves on each plant was measured using a leaf area meter (LI-1300, Licor, Lincoln, NE). Transpiration rate data were expressed on a plant leaf area basis.

All TR data were fit to a two-segment linear regression model in GraphPad Prism (v. 8.0. GraphPad Software Inc., San Diego, CA) similar to Shekoofa et al.^27^. Coefficients from this two-segment model were used to define two intersecting linear regressions; the VPD value at the breakpoint between the two linear segments was recorded as well as the slope of each segment^27^. When the slopes were not significantly different (α = 0.05) between the two segments, data were analyzed via simple linear regression in Prism. All data were subjected to Tukey’s Honestly Significant Difference (HSD) test at α = 0.05 in SAS (SAS Institute, Cary, NC).

### Effects of progressive soil drying

A series of greenhouse experiments was conducted to determine effects of progressive soil drying on foramsulfuron efficacy for goosegrass control. In all experiments, goosegrass was established in 5.7 L greenhouse pots filled with the same silt loam soil or silica sand used in our VPD experiments. Soil types were evaluated in separate experiments. Each pot was sown using ten goosegrass seeds placed at a 1 cm depth in the soil profile. Pots were placed inside a greenhouse at WTREC, irrigated daily, and maintained at air temperatures (day/night) of 32/25 °C. Once maturing to a three-tiller growth stage, each pot was sealed with two plastic bags (15.L. Glad Products Company. Oakland, CA). A plastic tube (13-mm-diam. × 126-mm-long) was also inserted to allow for controlled watering. All pots were weighed immediately after bagging and that measurement was recorded as the initial plant weight. Once weights were recorded, plants were treated with foramsulfuron (0 or 44 g ha^−1^) using previously described equipment. Treatments were replicated 10 times and arranged in a completely randomized design.

Every day the pots were weighed from 13:00 to 14:00. Daily transpiration was calculated as the difference in weight of each pot on successive days^27^. For each foramsulfuron treatment (0 or 44 g ha^−1^), four pots were maintained under well-watered conditions after herbicide treatment and six pots were subjected to progressive soil drying [i.e., stress (SS)]. The well-watered (WW) plants were irrigated daily to maintain a weight of no less that 200 g of their initial weight. Pots subjected to progressive soil drying were watered as needed to maintain daily net water loss of no more than 100 g^30^. This process continued over a 4-week period. Wilting scores (WS) were recorded daily on a 0 to 5 scale, with a score of 0 indicating no wilt and a score of 5 indicating complete kill.

Transpiration rate data were subjected to two normalizations, similar to Shekoofa et al.^27^. A daily transpiration ratio (DTR) was calculated for all pots subjected to progressive soil drying; this value compared the water loss of an individual pot to the average water loss of all four WW pots. Calculating a DTR minimized the influence of large variations in daily transpiration rate over time. For each plant, the DTR was then divided by the mean transpiration ratio during the first three days of the experiment, when the soil was still high in water content. This value was termed the normalized transpiration ratio (NTR) and its value during the initial wet phase of the dry-down cycle was centered on a value of 1.0. Calculating NTR values accounts for variation in transpiration ratio among individual plants. In all experiments, transpiration rate was assessed daily until NTR values measured ≤ 0.1, which was defined as the point at which transpirable soil water was exhausted.

Plant responses to soil drying are often assessed via measurements of transpirable soil water^31,32^. In our experiments, the total transpirable soil water available to each goosegrass plant was calculated as the difference between the initial and final weight of each pot. To track soil drying, the fraction of transpirable soil water (FTSW) was determined daily by calculating the difference between daily and final weight, divided by the total transpirable soil water.

All data were analyzed using a two linear-segment regression analysis in Prism (v. 8.0. GraphPad Software Inc., San Diego, CA). This regression analysis generated the FTSW threshold to initiate a decline in NTR. The 95% confidence interval of the threshold for each treatment was used to assess statistical differences^33^. Statistical analysis was completed in JMP Pro v.15 (SAS Institute, Cary, NC) with treatment means separated using Tukey's HSD test at α = 0.05.

## Results and discussion

### Soil moisture effects on herbicide efficacy

A significant year-by-soil moisture-by-herbicide interaction was detected in goosegrass control data (*P* < 0.0001); therefore, data from each year were analyzed and are presented separately. A significant soil moisture-by-herbicide interaction was detected in goosegrass control data collected in 2018. Regardless of herbicide treatment, goosegrass control measured ≤ 24% 36 DAT for plants maintained under moisture stress (< 12% VMC; Table 1). Interestingly, when soil moisture was increased to the 12 to 20% VMC, goosegrass control increased for all herbicides tested with overall control ranging from 49 to 95% (Table 1). When VMC increased to > 20%, improvements in goosegrass control were less pronounced. For example, when comparing the intermediate and high VMC treatments (i.e., 12 to 20% and > 20%), there were no statistically significant increases in goosegrass control for fenoxaprop, foramsulfuron, thiencarbazone-methyl + foramsulfuron + halosulfuron-methyl, and topramezone. In 2019, there was no soil moisture-by-herbicide interaction; however, goosegrass control varied due to the main effects of soil moisture (Table 2) and herbicide treatment (data not presented). Goosegrass control was greatest with herbicides applied to plants maintained at > 20% VMC and lowest with applications made to plants at < 12% VMC.

**Table 1:** Effect of a soil moisture-by-herbicide-interaction on goosegrass [*Eleusine indica* (L) Gaertn] control with postemergence herbicides 36 days after treatment in a glasshouse experiment conducted the University of Tennessee (Knoxville, TN) in November 2018.

Volumetric soil moisture content (%)^a^	Herbicide^b^	Rate (g ha^−1^)	Goosegrass control (%)
< 12	carfentrazone-ethyl + 2,4-D + mecoprop-p + dicamba	28 + 857 + 269 + 78	13
	fenoxaprop-ethyl	144	10
	foramsulfuron	44	14
	thiencarbazone-methyl + foramsulfuron + halosulfuron-methyl	22 + 45 + 67	20
	topramezone	24	24
12 to 20	carfentrazone-ethyl + 2,4-D + mecoprop-p + dicamba	28 + 857 + 269 + 78	49
	fenoxaprop-ethyl	144	65
	foramsulfuron	44	87
	thiencarbazone-methyl + foramsulfuron + halosulfuron-methyl	22 + 45 + 67	95
	topramezone	24	55
> 20	carfentrazone-ethyl + 2,4-D + mecoprop-p + dicamba	28 + 857 + 269 + 78	71
	fenoxaprop-ethyl	144	68
	foramsulfuron	44	87
	thiencarbazone-methyl + foramsulfuron + halosulfuron-methyl	22 + 45 + 67	93
	topramezone	24	48
LSD_0.05_			10

^a^Volumetric soil moisture content measured daily using moisture meter (ML-3 Theta Probe. Delta-T Devices. Cambridge, United Kingdom).

^b^Adjuvants were included with herbicides per label recommendations. Herbicides were applied in an enclosed spray chamber (Generation III track sprayer. DeVries Manufacturing, Hollandale, MN) using a water carrier at 215 L ha^−1^ through an 8004 EVS nozzle (TeeJet, Wheaton, IL). Application made 5 November 2018.

**Table 2:** Effect of volumetric soil moisture content on goosegrass [*Eleusine indica* (L) Gaertn] control 36 days after treatment with postemergence herbicides in a glasshouse experiment conducted Rutgers University (New Brunswick, NJ) in July 2019.

Volumetric soil moisture content (%)^a,b,c^	Goosegrass control (%)
< 12	23
12 to 20	40
> 20	53
LSD_0.05_	8

^a^Volumetric soil moisture content measured daily using moisture meter (ML-3 Theta Probe. Delta-T Devices. Cambridge, United Kingdom).

^b^Herbicides included carfentrazone-ethyl + 2,4-D-ester + mecoprop-p + dicamba (Speedzone. PBI Gordon Corporation. Shawnee, KS) at 28 + 857 + 269 + 78 g ha^−1^, respectively; topramezone (Pylex. BASF Corporation. Research Triangle Park, NC) at 24 g ha^−1^; fenoxaprop-ethyl (Acclaim Extra. Bayer Crop Science. Cary, NC) at 140 g ha^−1^; foramsulfuron (Revolver. Bayer Crop Science. Cary, NC) at 44 g ha^−1^; and thiencarbazone-methyl + foramsulfuron + halosulfuron-methyl (Tribute Total. Bayer Crop Science. Cary, NC) at 22 + 45 + 67 g ha^−1^, respectively. Adjuvants were included per label recommendations.

^c^Herbicides were applied in an enclosed spray chamber (Generation III track sprayer. DeVries Manufacturing, Hollandale, MN) using a water carrier at 215 L ha^−1^ through an 8004 EVS nozzle (TeeJet, Wheaton, IL). Application made 22 July 2019.

Our findings mirror those reported by other researchers exploring effects of soil moisture on grass weed control efficacy, particularly with herbicidal inhibitors of ACCase^12–14,16,19^. In 2018, the ACCase inhibiting herbicide fenoxaprop-ethyl controlled goosegrass 10% when applied to plants maintained at < 12% VMC compared to 68% when applied to plants at > 20% VMC (Table 1). This response was also observed with herbicidal inhibitors of ALS such as foramsulfuron and thiencarbazone-methyl + foramsulfuron + halosulfuron-methyl in this study. Xie et al.^34^ reported that moisture stress reduced phytotoxicity of the ALS inhibiting herbicide imazamethabenz-methyl applied to wild oat; however, the researchers also concluded that changes in herbicide absorption and translocation do not affect whole plant response. In 2019, this relationship between reduced soil moisture and compromised herbicide efficacy was observed regardless of herbicide as well (Table 2).

### Vapor pressure deficit and air temperature effects

**Figure 1:**
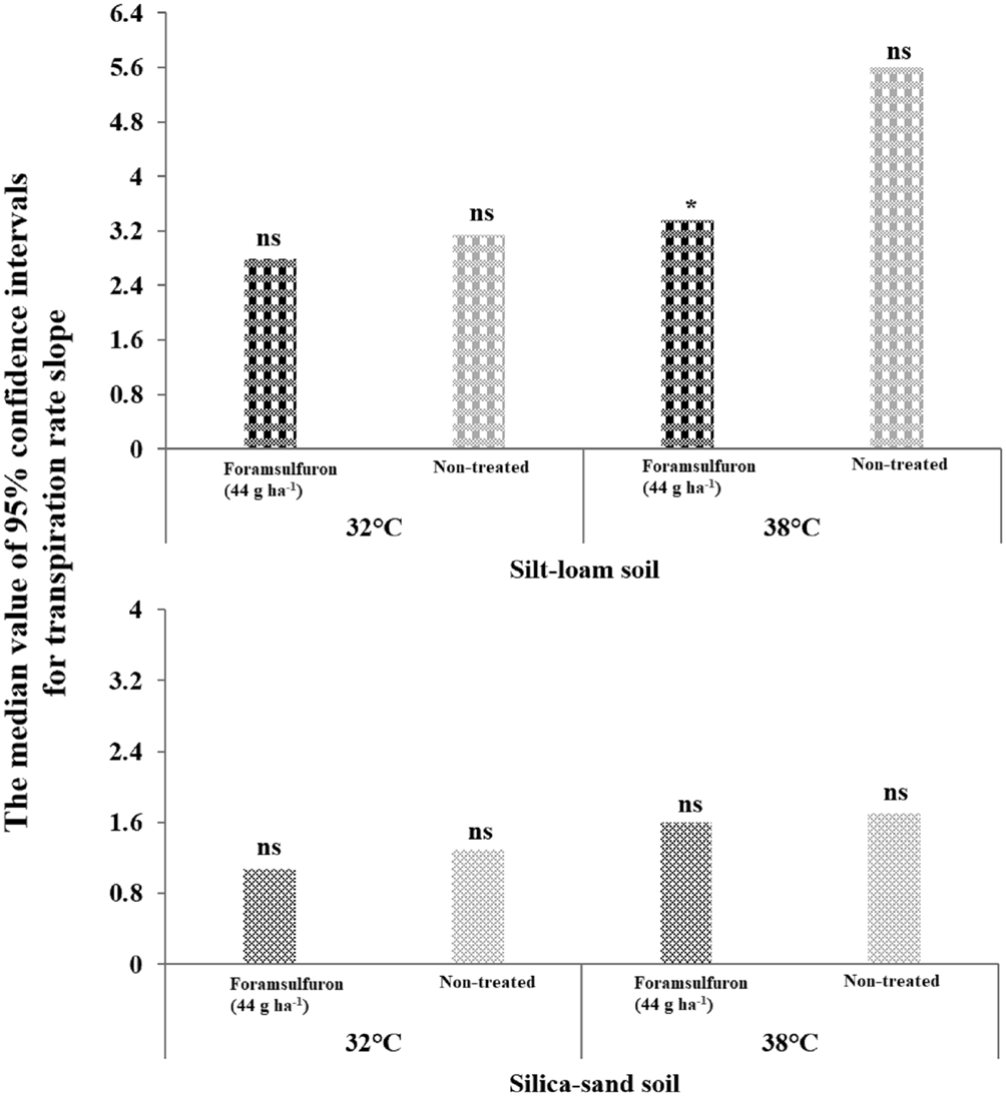
The median value of confidence intervals for transpiration rate slope among goosegrass [*Eleusine indica* (L) Gaertn] plants grown in silt-loam and silica-sand treated with foramsulfuron (0 or 44 g ha^−1^) under high vapor pressure deficit (> 3.0 kPa) at varying air temperatures in a walk-in growth chamber located at the West Tennessee AgResearch and Education Center (Jackson, TN) during 2019. Data were collected 14 days after treatment. Signifigance levels for each median confidence interval value are presented in the graph below (ns = non-significant, * = significant at α = 0.05).

Foramsulfuron was selected for use in VPD and air temperature experiments based on results of initial efficacy studies (Table 1). When foramsulfuron was applied to goosegrass grown in silt-loam soil under high VPD (> 3.0 kPa) and 38 °C temperature, there was a clear difference in transpiration rate compared to non-treated controls (Fig. 1). When evaporative demand was high (i.e., high VPD and air temperature), foramsulfuron reduced the transpiration rate compared to the non-treated, suggesting greater herbicidal  activity (Fig. 1). In other research, greater ambient air temperature improved mesotrione efficacy against common cocklebur (*Xanthium strumarium*) and velvetleaf (*Abutilon theophrasti* (L.) Medic.), which was attributed to increased herbicide absorption and translocation^35,36^. However, this relationship can vary among weed species as imazamethabenz-methyl provided optimal blackgrass (*Alopecurus myosuroides* Huds.) control at day/night temperatures of 26/16 °C, whereas optimal wild oat control was observed at 16/10 C^37^. In current study, leaf area of treated goosegrass plants grown under high VPD (> 3.0 kPa) and 38 °C temperature was significantly lower than non-treated controls, another indicator of greater herbicidal activity in conditions of high evaporative demand (Table 3). Leaf anatomy, leaf area, and leaf shape can be influenced by air and soil temperature, which, in turn, determine the time when plants are most susceptible to herbicides^38^. Our findings indicate that multiple environmental factors including temperature and VPD may have a significant impact on the efficacy of foramsulfuron for goosegrass control.

**Table 3:** Leaf area and tiller counts for goosegrass [*Eleusine indica* (L) Gaertn] plants treated with foramsulfuron (44 g ha^−1^) in vapor pressure deficit experiments at the West Tennessee Ag Research and Education Center (Jackson, TN) during 2019. Data collected from plants maintained at a vapor pressure deficit of > 3.0 kPa and air temperature of 38 °C.

Soil Type	Foramsulfuron rate	Leaf Area		Tillers	
		4 DAT	14 DAT	4 DAT	14 DAT
	g ha^−1^	mm^2^		# plant^−1^	
Silt loam	0	304	521	9.0	13.2
	44	302	308	10.8	11.0
*P* value		ns^a^	0.0012	ns	ns
Silica sand	0	129	100	7.0	7.2
	44	119	108	7.2	7.6
*P* value		ns	ns	ns	ns

^a^ns = not significant via Tukey’s Honestly Significant Difference Test at α = 0.05.

### Effects of progressive soil drying

**Figure 2:**
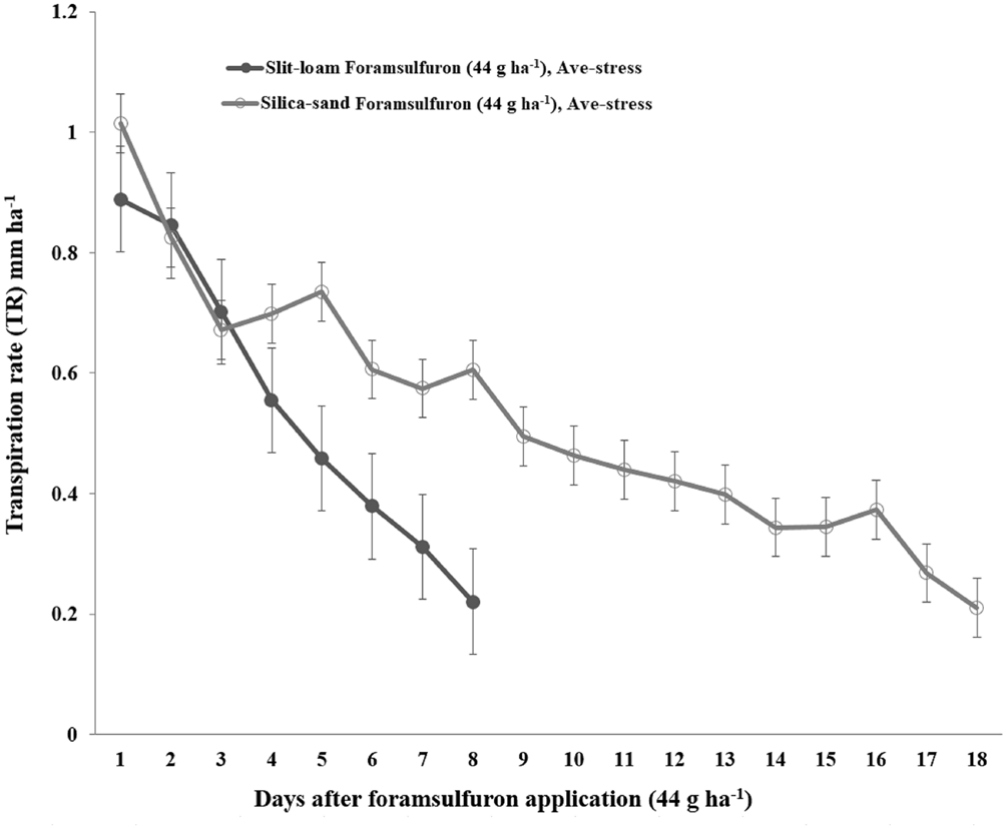
Transpiration rate (TR) data collected after treating three-tiller goosegrass [*Eleusine indica* (L) Gaertn] with foramsulfuron (0 or 44 g ha^−1^) in progressive soil drying experiments conducted in a greenhouse located at the West Tennessee AgResearch and Education Center (Jackson, TN) during 2019. Error bars represent standard error of the mean on each date.

Foramsulfuron applications to goosegrass growing in silt-loam reduced transpiration rate to 0.2 mmh^−1^ within eight days (Fig. 2). Comparatively, eighteen days were required for an equivalent drop in transpiration rate when goosegrass plants growing in silica-sand were treated with foramsulfuron (Fig. 2). Correspondingly, the average wilting score for treated goosegrass plants grown in silt-loam under dry down was higher (WS = 1.10, *P* < 0.05) than plants grown in silica-sand (WS = 0.3) eight days after foramsulfuron treatment.

**Figure 3:**
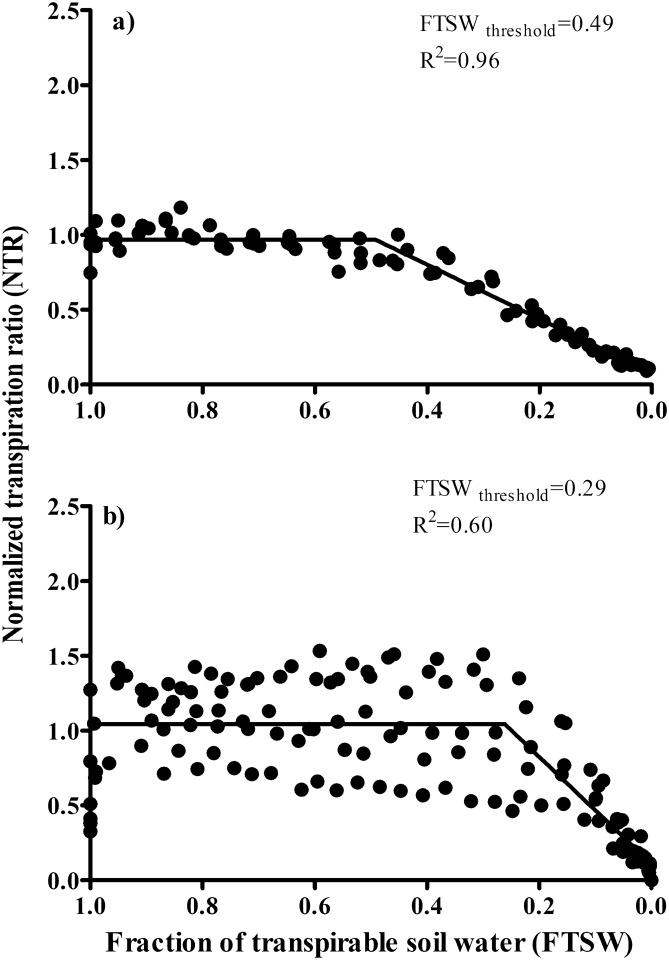
Normalized transpiration rate (NTR) of goosegrass [*Eleusine indica* (L) Gaertn] in silt-loam (**a**) and silica-sand (**b**) subjected to a drying cycle plotted against the fraction of transpirable soil water during progressive soil drying experiments conducted in a greenhouse located at the West Tennessee AgResearch and Education Center (Jackson, TN) during 2019.

In the absence of herbicide, normalized transpiration rate (NTR) versus fraction of transpirable soil water (FTSW) graphs for goosegrass plants grown in silt-loam (Fig. 3a) and silica-sand (Fig. 3b) illustrated large differences in the FTSW threshold between soil types (Fig. 3). There were also large differences in the response curves obtained based on the two expressions of extractable soil water (i.e., 0.49 vs. 0.29). Our study result reveals that the efficacy of foramsulfuron may vary by soil type (Fig. 2). On a finer textured soil (i.e., silt-loam), a high rate of transpiration can be sustained (Fig. 3) which can aid greater herbicide efficacy shortly after application of foramsulfuron (Fig. 2) compared to coarse textured soil (i.e., silica-sand). Soil texture strongly mediates plant water availability through its control on plant-soil hydraulic characteristics^39^.

## Conclusion

Results indicate that environmental conditions at application greatly affect the efficacy of herbicides for postemergence goosegrass control. An array of herbicides varying in mode of action, but particularly foramsulfuron, provided greater goosegrass control when soil moisture before, during, or after the herbicide application did not limit goosegrass growth. Controlled environment research indicated that conditions of high evaporative demand (i.e., elevated air temperature and VPD > 3.0 kPa) coupled with a greater fraction of soil water available for transpiration in the days following foramsulfuron treatment are important to optimize herbicide activity. Future research to better understand the mechanisms affecting foramsulfuron performance under varying levels of evaporative demand is warranted. Overall, our findings suggest turfgrass managers should maintain adequate soil moisture, particularly in sandy soils, around the time of herbicide application to optimize goosegrass control provided by postemergence herbicides.

## Data Availability

The datasets generated and analyzed during this research are available from the corresponding author upon reasonable request.
